# High-Temperature Performance of Metakaolin-Based Geopolymer Recycled Mortar with Pumice Powder

**DOI:** 10.3390/ma19183819

**Published:** 2026-09-08

**Authors:** Xudong Zhu, Feifei Jiang, Changwei Chen, Hui Liu, Pinghua Zhu, Yang Li, Tianyu Ma

**Affiliations:** 1 School of Civil Engineering, Nantong Institute of Technology, Nantong 226002, China; 2 School of Urban Construction, Changzhou University, Changzhou 213164, China; 3 Jiangsu Xiechuang Traffic Construction Co., Ltd., Changzhou 213119, China

**Keywords:** metakaolin-based geopolymer, recycled fine aggregate, pumice powder, residual mechanical properties, thermal damage mechanism

## Abstract

This study investigates metakaolin-based geopolymer recycled mortar (GRM) in which recycled fine aggregate (RFA) was volumetrically replaced by pumice powder (PP) at 0% and 60–100%. Ambient compressive and tensile bond strengths were measured, followed by exposure to 600–800 °C for 1–3 h. The evaluation encompassed mass loss ratio, residual mechanical properties, temperature sensitivity, micro-phase evolution, and strength-normalized carbon intensity. Results demonstrated that PP replacement produced a non-monotonic response, governed by the trade-off between improved particle packing and the depletion of the rigid granular skeleton. Among the PP-containing mixtures, PP_70_ (70% replacement) showed a comparatively favorable mechanical response, with an ambient compressive strength of 31.7 MPa and a mean residual compressive strength of 18.8 MPa (59.3% of its initial value) after exposure to 800 °C for 3 h. Notably, tensile bond strength exhibited greater temperature sensitivity than compressive strength, with deterioration accelerating significantly above 700 °C. SEM and XRD analyses elucidated this macroscopic divergence via a two-stage damage mechanism: while dehydration and matrix contraction dominated at 600 °C, prolonged exposure at 800 °C induced structural rearrangement, with the dominant damage becoming increasingly concentrated at the RFA–matrix interface. Although substituting RFA with processed PP inherently increased the absolute embodied carbon, PP_70_ exhibited the lowest strength-normalized carbon intensity among the modified mixtures after exposure to 800 °C for 3 h. These findings indicate that, among the investigated high-volume PP mixtures, PP_70_ provided a comparatively favorable compromise between thermal-mechanical performance and environmental cost.

## 1. Introduction

The construction industry faces increasing pressure to reduce greenhouse-gas emissions, conserve natural resources, and reutilize construction and demolition waste [1,2,3]. Alkali-activated and geopolymer binders have received considerable attention because they can reduce dependence on clinker-based cement by converting aluminosilicate precursors into hardened inorganic networks [4,5]. In parallel, recycled aggregates provide a practical route for returning demolished concrete to the construction-material cycle. Combining these two strategies in geopolymer recycled mortar (GRM) may therefore offer both resource-conservation and waste-management benefits. However, these benefits cannot be inferred solely from the absence of Portland cement, because the production of alkaline activators, particularly sodium silicate and sodium hydroxide, may contribute substantially to the embodied carbon of geopolymer materials [6]. Moreover, recycled fine aggregate (RFA) generally contains adhered old mortar, pre-existing defects, and inherited interfacial transition zone (ITZ), resulting in higher water absorption and greater heterogeneity than natural aggregate [7,8]. These characteristics affect moisture distribution, interfacial stress transfer, and the continuity of the granular load-bearing skeleton [9,10]. Consequently, the thermal response of GRM cannot be directly extrapolated from that of geopolymer paste or mortar containing natural aggregate.

Geopolymers are considered promising for heat-resistant applications because their cross-linked aluminosilicate network differs from the calcium-rich hydration products of Portland-cement materials [11,12]. Nevertheless, they are not immune to thermal deterioration. Heating causes evaporation of free and physically bound water, dehydration and dehydroxylation of reaction products, shrinkage of the geopolymer gel, and redistribution of internal stresses [13]. At higher temperatures, structural rearrangement or partial crystallization may occur. In mortar, these transformations interact with differential thermal deformation among the binder, aggregate and ITZ, thereby promoting pore coarsening and interfacial separation [14]. Porous RFA can further complicate this process because adhered old mortar alters moisture release and local deformation. Previous studies have shown that compressive strength alone cannot fully represent the thermal response of geopolymer composites, because tensile and bond-related properties may deteriorate more rapidly when local cracks form preferential fracture paths [15,16]. Most investigations have focused on binder chemistry or target temperature, with less attention given to how aggregate modification affects the transition from moisture-related damage to matrix and interface deterioration.

Pumice is a porous volcanic aluminosilicate that has been investigated as a lightweight aggregate, supplementary cementitious material, and precursor in alkali-activated systems [17]. Existing studies have shown that finely ground pumice can participate in alkaline reactions and modify the pore structure of the resulting binder [18]. Most previous investigations have treated pumice powder (PP) as part of the reactive binder or as a conventional cement-replacement material [19]. These findings cannot be directly transferred to a system in which PP volumetrically replaces RFA, because this substitution simultaneously changes the particle packing and structure of the particle–matrix interfaces. RFA covers the fine aggregate size range of 0.075–4.75 mm, whereas PP belongs predominantly to the powder fraction. Therefore, increasing the PP replacement level simultaneously modifies the overall particle-size distribution, total solid surface area, powder-to-aggregate ratio, phase-volume distribution, and continuity of the granular load-bearing skeleton. Consequently, the effects associated with PP replacement should be understood as the combined result of material substitution and granulometric restructuring rather than solely as an intrinsic material effect of pumice powder. Replacing part of the highly absorptive RFA with fine PP may reduce the amount of water stored in adhered old mortar and improve the packing of the solid constituents. Conversely, extensive replacement decreases the volume and continuity of the rigid aggregate skeleton and increases the powder-dominated fraction, potentially impairing stress transfer and increasing the proportion of incompletely reacted particles or weak particle boundaries.

On this basis, PP replacement is hypothesized to influence the high-temperature performance of GRM through two competing effects: modification of the matrix and RFA-related moisture environment, and progressive weakening of the granular load-bearing skeleton. The first effect may reduce moisture accumulation and local structural heterogeneity while improving particle packing; the second may reduce aggregate restraint and facilitate crack localization when the PP content becomes excessive. This competition is expected to affect bulk compression and tensile load transfer differently because these properties are controlled by different damage scales. Mass loss can provide an indication of temperature-induced volatile release associated mainly with water removal and dehydroxylation, whereas residual compressive strength reflects the integrity of the bulk load-bearing structure. Tensile bond strength provides a more sensitive measure of the continuity of the bonded region and the development of preferential fracture paths. Microstructural and phase characterization can further provide supporting evidence for interpreting whether macroscopic degradation is associated primarily with matrix cracking, interfacial separation, or thermally induced rearrangement of the aluminosilicate structure. These indicators should therefore be considered collectively rather than as independent criteria.

Previous studies have separately investigated the high-temperature response and interfacial behavior of geopolymer materials [13,14,15,16] and the incorporation of pumice in cementitious and alkali-activated systems [17,18,19]. However, the effect of volumetrically replacing RFA with PP on the coupled moisture-release, deformation, and damage evolution of GRM under elevated temperatures has not yet been systematically established. The main objective of this study is to determine how high-volume volumetric replacement of RFA by PP affects the thermal-mechanical stability and damage evolution of metakaolin-based GRM. In addition to the PP_0_ reference mixture, PP replacement levels of 60–100 vol.% were selected so that PP replaced the majority of the original RFA volume, thereby enabling the feasibility and performance limits of a substantially powder-modified granular system to be examined. The ambient compressive and tensile bond strengths are first determined, after which the specimens are exposed to 600, 700, and 800 °C for 1, 2, and 3 h. Mass loss ratio, residual compressive strength, and residual tensile bond strength are evaluated, and their relationships and temperature sensitivities are quantified. Scanning electron microscopy and X-ray diffraction are used to examine the thermal evolution. Finally, embodied carbon and strength-normalized carbon intensity are calculated to assess whether the thermal-mechanical effects of PP replacement are accompanied by an acceptable environmental cost.

## 2. Materials and Methods

### 2.1. Raw Materials

**Figure 1:**
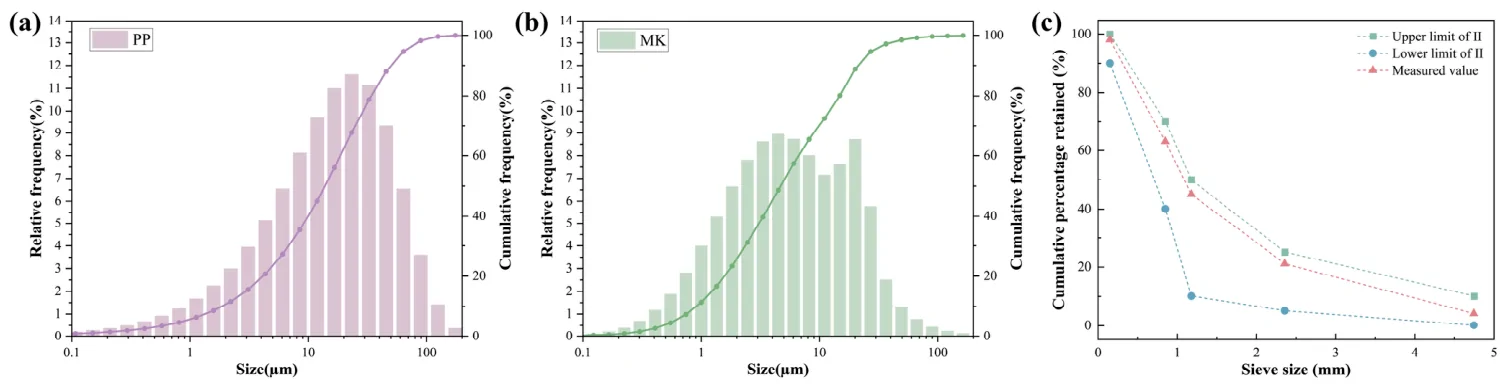
Particle-size distributions of (**a**) PP, (**b**) MK, and (**c**) RFA.

Metakaolin (MK) was used as the aluminosilicate precursor, produced by Gongyi Oushang Refractory Co. Ltd. in China. RFA, with a particle-size range of 0.075–4.75 mm, was obtained from Jiangsu Lvhe Environmental Technology Co., Ltd., (Changzhou, China). The apparent density, water absorption, and fineness modulus of the RFA were 2273 kg/m^3^, 9.4%, and 2.9, respectively. Pumice powder (PP) was an industrial by-product produced by Hebei Runhuabang New Materials Technology Co., Ltd., (Shijiazhuang, China). The particle-size distributions of MK and PP were determined using a laser particle-size analyzer (ZEN3600, Malvern Panalytical Ltd., Malvern, UK). The particle-size distribution of RFA was determined by sieve analysis. The particle size distribution is shown in Figure 1. The chemical composition of MK and PP was determined using X-ray fluorescence spectroscopy(Epsilon 4, Malvern Panalytical Ltd., Malvern, UK). The relevant results are presented in Table 1. Their physical properties are presented in Table 2. The alkaline activator consisted of sodium hydroxide (NaOH) solution and commercial sodium silicate solution. The NaOH solution had a concentration of 10 mol/L and was prepared 24 h before mixing and allowed to cool to room temperature. The sodium silicate solution contained 21.5 wt.% SiO_2_, 18.5 wt.% Na_2_O, and 60 wt.% H_2_O, corresponding to a silica modulus (Ms = SiO_2_/Na_2_O molar ratio) of 1.2.

### 2.2. Mixture Proportions and Specimen Preparation

The present study focuses specifically on the feasibility and performance limits of high-volume PP incorporation. Accordingly, PP was used to replace RFA on an equal absolute-volume basis at replacement levels of 0%, 60%, 70%, 80%, 90%, and 100%. The mixtures were designated PP_0_, PP_60_, PP_70_, PP_80_, PP_90_, and PP_100_, respectively, where the number denotes the volumetric replacement percentage of RFA by PP. The mixture proportions of pumice powder modified geopolymer recycled mortar (PP-GRM) are summarized in Table 3. Before mixing, the RFA was pre-wetted based on the water absorption rate to minimize the preferential absorption of the alkaline activator. MK, RFA, and PP were initially dry-mixed for 1 min. The well-mixed alkaline activator solution (sodium hydroxide, sodium silicate) was added sequentially, and mixing was continued until a homogeneous mortar was obtained. The fresh mortar was placed into the moulds in two layers and compacted on a vibrating table. Cubic specimens with dimensions of 50 × 50 × 50 mm^3^ were prepared for the compressive-strength and elevated-temperature tests. Samples with dimensions of 40 × 40 × 6 mm^3^ were used for the test of tensile bond strength. After casting, the specimens were sealed and cured at 70 °C for 24 h. They were subsequently demoulded and stored under standard curing conditions (20 ± 2 °C, RH ≥ 95%) until an age of 7 days. At 7 days, specimens designated for the ambient reference condition were subjected to the same 50 °C drying treatment as the thermally exposed specimens and tested after cooling. Specimens designated for elevated-temperature exposure were transferred to a drying oven at the age of 7 days.

### 2.3. Elevated-Temperature Exposure

**Figure 2:**
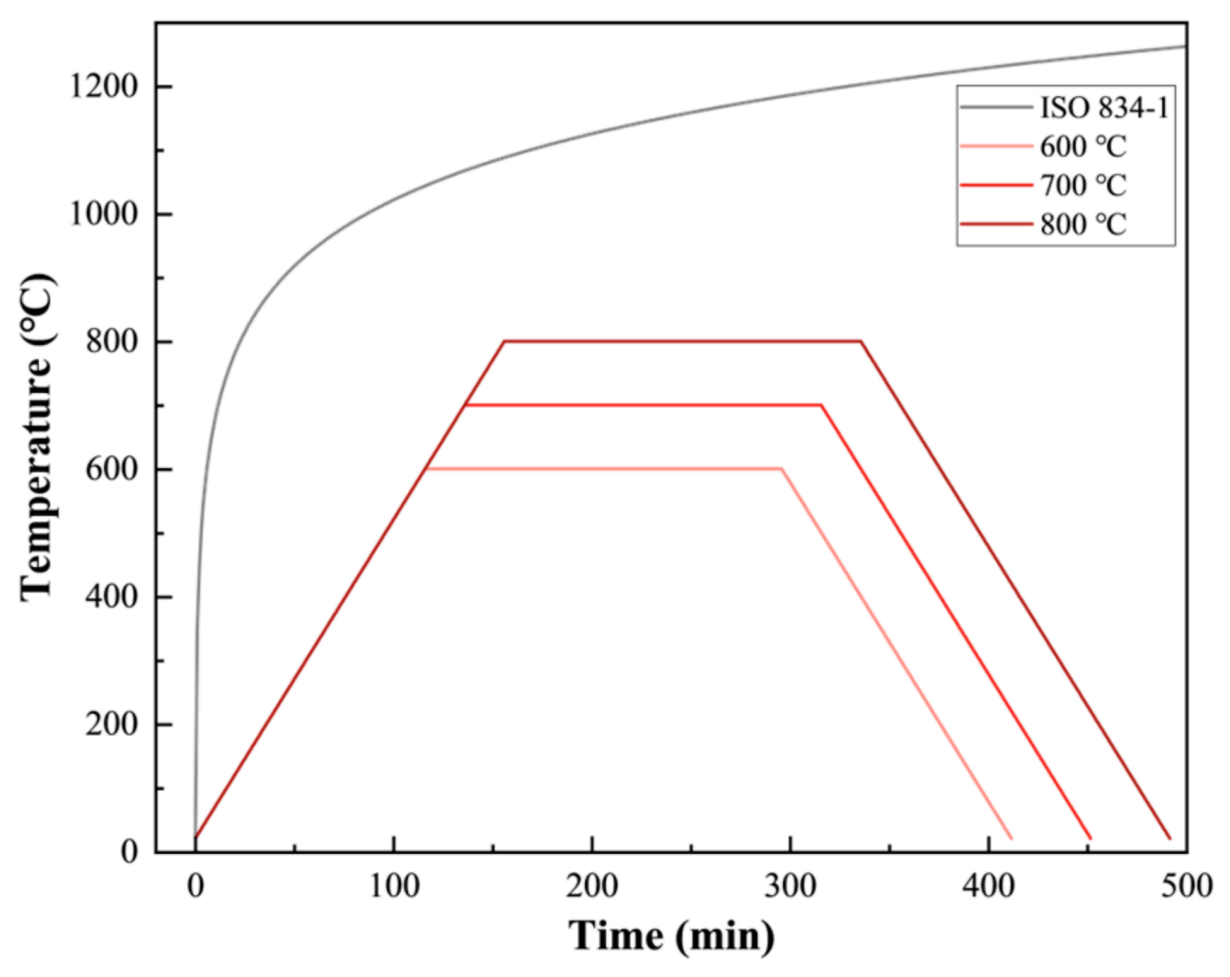
Heating and cooling regimes used for the elevated-temperature exposure.

Before thermal exposure, the specimens were dried at 50 °C until a constant mass was achieved and then cooled to room temperature. Their initial masses were recorded before heating. The specimens were heated to target temperatures of 600, 700, and 800 °C using the heating program shown in Figure 2. After the target temperature was reached, it was maintained for 1, 2, or 3 h. The furnace was then switched off, and the specimens were allowed to cool naturally inside the furnace. The heating program was designed with reference to ISO 834-1 [20]. After the target temperature and time was reached, the furnace was then switched off, and the specimens were allowed to cool naturally inside the furnace to minimize additional thermal shock. After cooling to room temperature, the specimens were visually examined for cracking, spalling, and changes in surface appearance. Their properties were subsequently determined.

### 2.4. Test Methods

#### 2.4.1. Mass Loss Ratio

The specimens were dried at 50 °C until the difference between two successive mass measurements was less than 0.2%. After cooling in a desiccator, their dry masses were measured. The mass loss ratio after elevated-temperature exposure was calculated using Equation (1):

(1) $$W=\frac{m_{0}-m_{T}}{m_{0}}\times 100\%$$

where *W* represents the mass loss ratio; *m*_0_ is the specimen mass before heating; *m_T_* is the mass after exposure to temperature (*T*) and subsequent cooling to room temperature.

#### 2.4.2. Compressive and Tensile Bond Strength

The compressive strength and tensile bond strength were measured using an electro-hydraulic servo universal testing machine (TYA-300B, Wuxi Xinluda Instrument Equipment Co., Ltd., Wuxi, China) at a loading rate of 0.2 MPa/s. The compressive strength was conducted with reference to the procedure specified in JGJ/T 70–2009 [21], with modifications in the number of replicates and loading protocol. Cubic specimens (50 × 50 × 50 mm^3^) were used to measure compressive strength and were not intended to represent the specimen geometry specified in the standard. The final results were reported as the arithmetic mean of triplicate measurements.

Tensile bond strength was evaluated using a modified laboratory direct-tension test. Free-standing geopolymer mortar specimens with dimensions of 40 × 40 × 6 mm were prepared. After curing, steel loading fixtures were bonded to the two opposite 40 × 40 mm faces of each specimen using epoxy resin. After complete curing of the adhesive, the specimen was mounted in the universal testing machine at a loading rate of 0.2 MPa/s and subjected to tensile loading normal to the bonded faces until failure. Three replicate specimens were tested for each condition, and the mean value was reported.

#### 2.4.3. Correlation and Temperature-Sensitivity Analysis

Simple linear regression was used to evaluate the relationships among mass loss ratio, residual compressive strength, and residual bond strength. The general regression model was expressed as:

(2) y=ax+b

where a and b are the slope and regression intercept, respectively. The strength of each relationship was evaluated using the coefficient of determination *R*^2^. The results were interpreted as descriptive associations among the measured properties rather than evidence of direct causality.

To quantify the response of the GRM to increasing exposure temperature, temperature sensitivity indices were calculated separately for the residual compressive strength, residual bond strength, and mass loss ratio. At a fixed pumice powder replacement level and exposure duration, the average temperature sensitivity indices of the residual compressive strength and bond strength over the range of 600–800 °C were determined as follows:

(3) $$S_{c}=\frac{f_{c,800}-f_{c,600}}{\left(800-600\right)/100}$$

(4) $$S_{b}=\frac{f_{b,800}-f_{b,600}}{\left(800-600\right)/100}$$

where *f_c_*_,600_ and *f_c_*_,800_ are the residual compressive strengths after exposure at 600 and 800 °C (MPa), respectively, and *f_b_*_,600_ and *f_b_*_,800_ are the corresponding residual bond strengths (MPa). The units of *S_c_* and *S_b_* are MPa per 100 °C. Negative values indicate reductions in residual strength with increasing temperature, while a smaller absolute value, i.e., a value closer to zero, indicates lower temperature sensitivity.

The temperature-induced increase in mass loss was evaluated by:

(5) $$S_{m}=\frac{M_{800}-M_{600}}{\left(800-600\right)/100}$$

where *M*_600_ and *M*_800_ are the mass loss ratio measured after exposure at 600 and 800 °C (%), respectively. The unit of (*S_m_*) is percentage points per 100 °C. A smaller *S_m_* indicates a slower increase in mass loss ratio with increasing temperature.

Because the response rate may differ between successive temperature intervals, the stagewise relative change in each property was additionally calculated as:

(6) $$R=|\frac{X_{T_{1}}-X_{T_{2}}}{X_{T_{1}}}|\times 100\%$$

where *R* represents relative reduction in mechanical performance (%); *X* denotes the residual compressive strength, residual direct tensile strength, or mass loss ratio; *T*_1_ and *T*_2_ denote two consecutive exposure temperatures. The values were calculated separately for the 600–700 °C and 700–800 °C intervals.

#### 2.4.4. Microstructural Characterization

The microstructures of selected specimens were examined using scanning electron microscopy (TESCAN MIRA SEM, Brno, Czech Republic) at an accelerating voltage of 10 kV, a working distance of 8.5 mm, and a spot size of 50 nm. The micrographs were acquired using a secondary-electron (SE) detector (SmartLab, Rigaku, Tokyo, Japan). Representative fragments were collected from the interior of the specimens and from the geopolymer matrix interfacial region. Before observation, the samples were solvent-exchanged with absolute ethanol, dried under vacuum, and coated with a conductive layer.

#### 2.4.5. Phase Composition

The phase compositions were characterized using X-ray diffraction (SmartLab, Rigaku, Tokyo, Japan; Cu Kα, 40 kV/40 mA) with Cu Kα radiation. Representative samples were ground and passed through a 75 μm sieve. The diffraction patterns were collected over a 2θ range of 5–80° at a scanning rate of 5 °/min. The crystalline phases were identified by matching the diffraction peaks with reference patterns from the ICDD PDF-4+ database.

## 3. Results and Discussion

### 3.1. Ambient-Temperature Mechanical Properties

**Figure 3:**
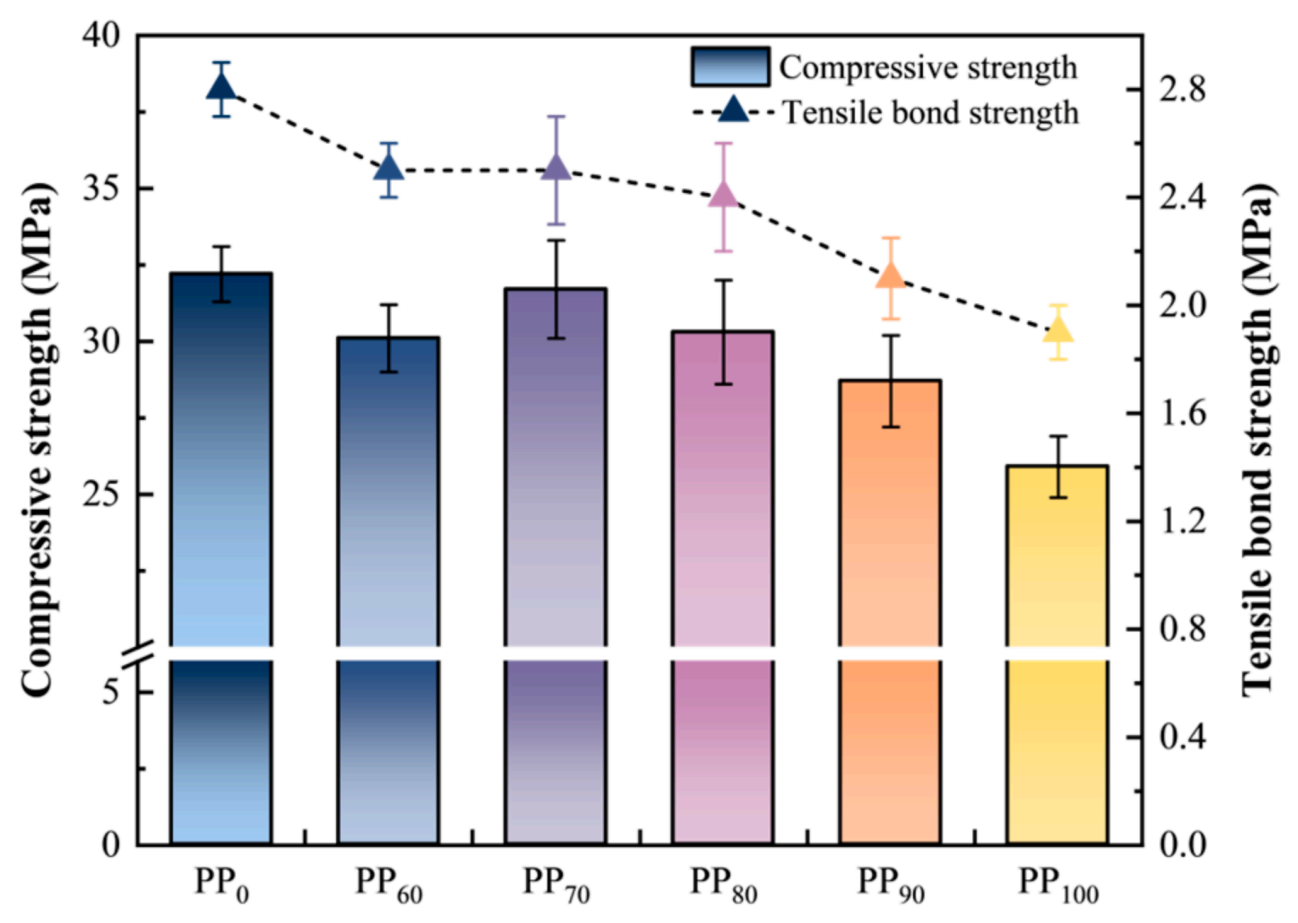
Ambient-temperature compressive and tensile bond strength.

Figure 3 presents the ambient-temperature compressive and tensile bond strengths of the PP-GRM mixtures. The compressive strength exhibited a non-monotonic dependence on the volumetric replacement of RFA by PP. PP_70_ exhibited the highest compressive strength (31.7 MPa) among the PP-containing mixtures, exceeding that of PP_60_ (30.1 MPa) by 5.3% and remaining only 1.6% lower than that of PP_0_ (32.2 MPa). Increasing the PP content shifts the solid system toward a finer particle population, increases the powder-to-aggregate ratio and total particle surface area, and simultaneously reduces the volume and continuity of the RFA load-bearing skeleton. Within the investigated series, PP70 appears to provide a comparatively favorable balance between the potential filling contribution of the fine PP particles and the preservation of a sufficient granular skeleton. While its Si- and Al-bearing components may contribute to the formation of additional aluminosilicate reaction products [22]. However, when the PP replacement level exceeded 70%, the progressive depletion of the RFA skeleton and the increasingly powder-dominated structure outweighed the potential beneficial effects. Moreover, the substantial increase in the powder fraction at high PP contents may have limited the effective dissolution and reaction of the solid constituents, thereby increasing the proportion of unreacted particles and pore defects [23]. These combined effects account for the marked strength reduction observed for PP_90_ and PP_100_.

In contrast to the compressive strength, the tensile bond strength decreased almost monotonically with increasing PP replacement. The tensile bond strength decreased from 2.8 MPa for PP_0_ to 2.5 MPa for both PP_60_ and PP_70_. Compared with PP_0_, the tensile bond strength of PP_100_ decreased by 32.1%. This trend indicates that tensile bond performance was more sensitive than compressive strength to the progressive replacement of RFA. Although PP_60_–PP_70_ may provide a comparatively favorable balance between the fine-particle contribution and the remaining granular skeleton, further increasing PP replacement to 80–100% reduces the contribution of the rigid aggregate skeleton and may impair structural continuity [24]. Overall, among the investigated PP-containing mixtures, PP_70_ showed a relatively favorable combination of compressive and direct tensile strengths.

### 3.2. Surface Appearance After Elevated-Temperature Exposure

**Figure 4:**
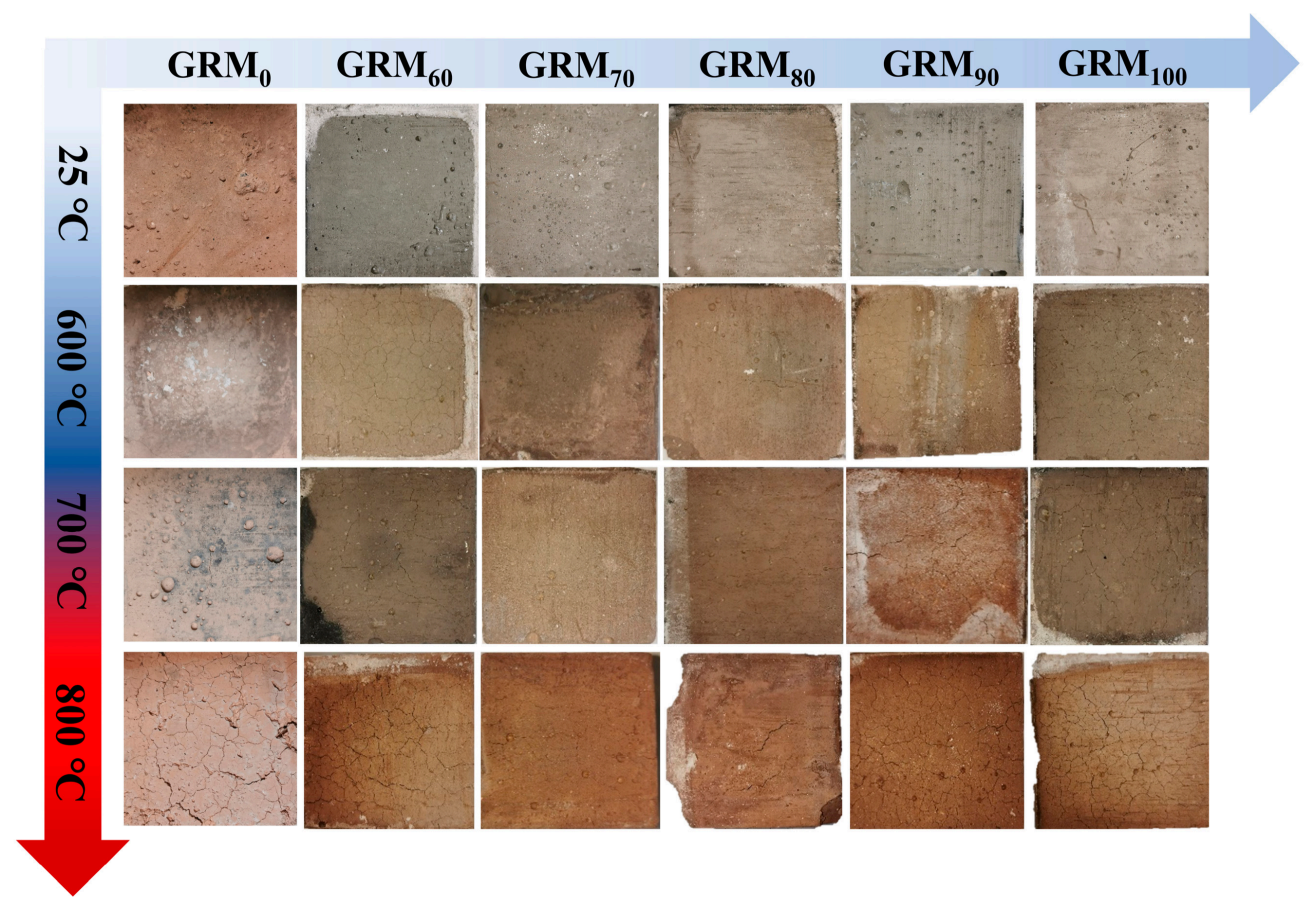
Macroscopic appearance of the GRM specimens after 3 h of exposure at different temperatures.

Figure 4 presents the macroscopic appearance of the specimens after 3 h of thermal exposure. The longer exposure duration was selected to provide a consistent comparison of representative surface deterioration at different temperatures. At 25 °C, all specimens exhibited intact surfaces without visible cracking or spalling, although differences in surface color were observed owing to the variation in PP content.

After exposure to 600 °C, the specimens generally retained their original geometry, and no apparent macrocracking or explosive spalling was observed. Localized darkening and surface streaks appeared on several specimens, indicating the onset of thermally induced moisture migration and changes in the geopolymer matrix. When the exposure temperature increased to 700 °C, the surface color progressively changed from gray to brown, accompanied by increased surface heterogeneity and localized roughening. The reddish-brown region observed on PP_90_ suggests that the thermal response was not spatially uniform throughout the specimen surface. These changes can be associated with the continued removal of chemically bound water and thermally induced changes in Fe-bearing constituents [25]. Despite the more pronounced discoloration, most specimens remained macroscopically intact at 700 °C with no extensive crack networks. At 800 °C, the deterioration became markedly more severe. Dense interconnected surface cracks were visible on PP_0_ and PP_60_, while PP_80_ and PP_100_ also developed pronounced surface cracking together with localized edge damage. In comparison, PP_70_ retained relatively complete surfaces. The development of cracking at 800 °C can primarily be attributed to the accumulation of thermal stresses caused by the differential deformation of the matrix, RFA, and PP particles [26].

The severity of macroscopic damage increased mainly with exposure temperature, while no consistent relationship was observed between PP replacement level and surface deterioration. Within the investigated series, PP_70_ exhibited comparatively limited visible surface damage under severe thermal exposure, although no strictly monotonic relationship between PP replacement and macroscopic deterioration was observed. At the upper replacement levels of 80–100%, the substantially reduced RFA content and increasingly powder-dominated structure may increase susceptibility to matrix shrinkage cracking and localized damage.

### 3.3. Mass Loss Ratio

**Figure 5:**
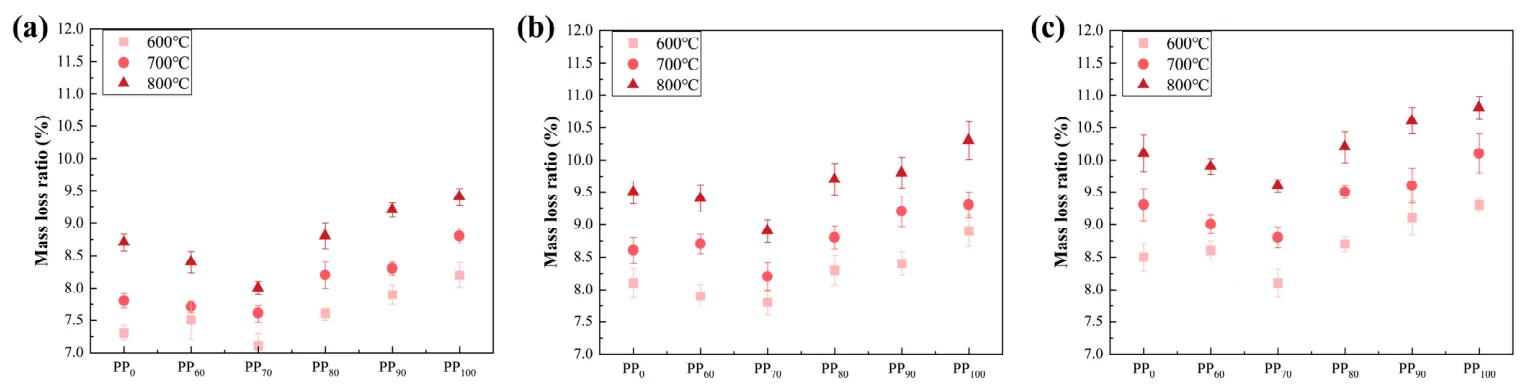
Mass loss ratio of PP-GRM specimens after elevated-temperature exposure for different holding times: (**a**) 1 h, (**b**) 2 h, and (**c**) 3 h.

Figure 5 shows the mass loss ratio of the GRM specimens after exposure to 600, 700, and 800 °C for 1, 2, and 3 h. For all mixtures, the mass loss ratio increased with both exposure temperature and holding time, with the effect of prolonged exposure becoming more pronounced at higher temperatures. For example, the mass loss ratio of PP_70_ increased from 7.1% to 8.1% at 600 °C when the holding time was extended from 1 to 3 h. This behavior suggests that prolonged exposure facilitated heat penetration into the specimen interior and allowed more complete dehydration and dehydroxylation of the aluminosilicate reaction products [27]. At 800 °C, further condensation and structural rearrangement of the geopolymer gel may also have contributed to the increased mass loss ratio [28]. At each temperature and holding time, the mass loss ratio initially decreased as the PP replacement level increased from 0% to 70%, and subsequently increased at higher replacement levels. PP_70_ consistently exhibited the lowest mass loss ratio among the investigated mixtures.

The relatively low mass loss ratio of PP_70_ may be associated with the combined effects of replacing the highly absorptive RFA and improving the packing of the solid constituents. An appropriate PP content may reduce the amount of moisture retained within the aggregate and interfacial regions while producing a more compact matrix with fewer interconnected transport paths [29]. However, when the PP replacement exceeded 70%, the substantial reduction in RFA content weakened the granular skeleton and markedly increased the proportion of fine powder in the mixture. Excessive PP may also have increased the area of incompletely reacted particles and particle–matrix interfaces, facilitating moisture release and the development of thermally induced defects. Mass loss ratio primarily reflects the release of volatile components and should not be used alone to assess mechanical deterioration. Its relationship with high-temperature damage is therefore further examined through the residual compressive and tensile bond strengths in the following sections.

### 3.4. Residual Compressive Strength

**Figure 6:**
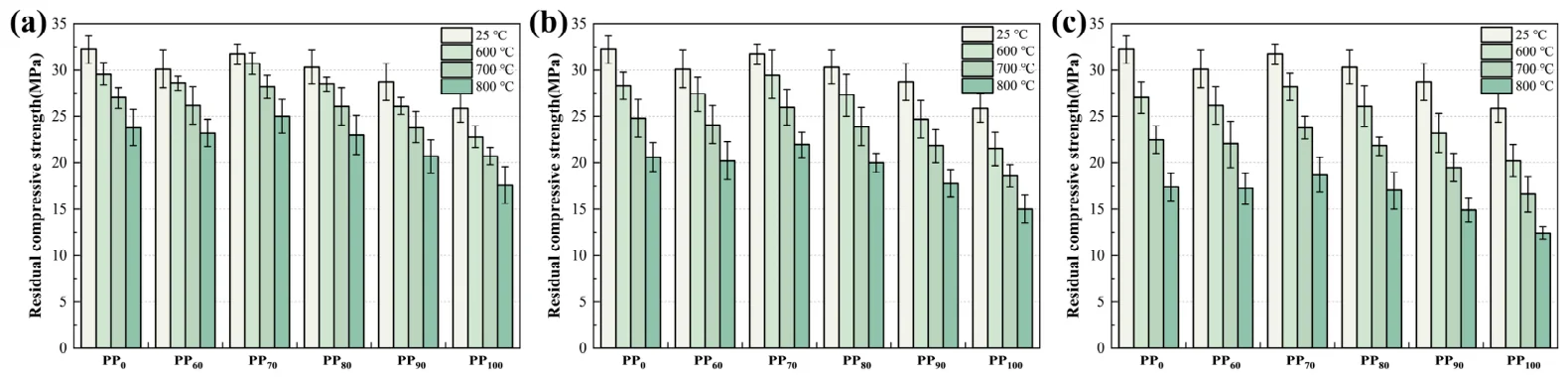
Residual compressive strength of PP-GRM specimens after exposure to 600, 700, and 800 °C for (**a**) 1 h, (**b**) 2 h, and (**c**) 3 h.

Figure 6 presents the residual compressive strengths of the PP-GRM specimens after exposure to 600, 700, and 800 °C for different holding times. Regardless of the PP replacement level, the residual compressive strength decreased progressively with increasing exposure temperature and holding time.

The influence of holding time became more pronounced at higher temperatures. For PP_70_, extending the holding time from 1 to 3 h reduced the residual compressive strength from approximately 30.7 to 28.2 MPa at 600 °C. In contrast, the strength decreased from approximately 25.0 to 18.8 MPa at 800 °C over the same period. This difference indicates that prolonged exposure intensified the thermal deterioration once a sufficiently high temperature was reached. Accordingly, exposure temperature was the principal factor governing strength degradation, while holding time amplified the damage, particularly at 700 and 800 °C.

The effect of PP replacement was consistent under the different thermal regimes. At each exposure temperature and holding time, the residual compressive strength initially increased with increasing PP replacement and reached its maximum at a replacement level of 70%, after which it decreased. Across the investigated thermal conditions, PP_70_ generally showed the highest mean residual compressive strength among the PP-containing mixtures. After exposure to 800 °C for 3 h, the mean residual compressive strength of PP_70_ was approximately 18.8 MPa, compared with 17.5 MPa for PP_0_ and 12.5 MPa for PP_100_. The corresponding strength-retention ratios were approximately 59.3%, 54.3%, and 48.3%, respectively. These results indicate that PP_70_ tended to maintain a relatively high mean residual compressive capacity under the investigated thermal conditions.

The superior residual performance of PP_70_ may result from a balance between matrix densification and thermally induced degradation. Under relatively moderate thermal exposure, further condensation and structural rearrangement of the aluminosilicate network may locally densify the matrix and partially offset the damage caused by dehydration [30]. An appropriate PP content may also improve particle packing and provide internal space for accommodating vapor migration and thermal deformation [25]. These effects are consistent with the comparatively low mass loss ratio of PP_70_ discussed in Section 3.3. However, as the temperature and holding time increased, the shrinkage of the geopolymer gel and differential thermal deformation among the matrix, RFA, and PP particles promoted microcrack propagation, resulting in a continuous loss of strength. This interpretation is further supported by the SEM observations presented in Section 3.7.

### 3.5. Residual Tensile Bond Strength

**Figure 7:**
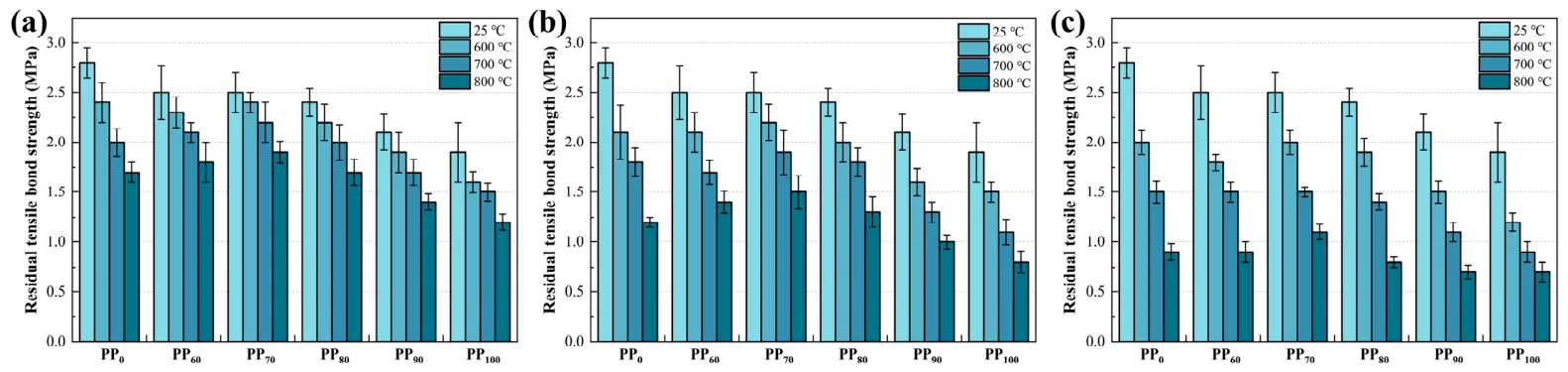
Residual tensile bond strength of PP-GRM specimens after exposure to 600, 700, and 800 °C for (**a**) 1 h, (**b**) 2 h, and (**c**) 3 h.

Figure 7 presents the tensile bond strengths of the PP-GRM specimens before and after exposure to 600, 700, and 800 °C for different holding times. For all mixtures, the tensile bond strength decreased with increasing exposure temperature and holding time. After 1 h of exposure, the bond-strength values ranged from 1.6 to 2.4 MPa at 600 °C, from 1.5 to 2.2 MPa at 700 °C, and from 1.2 to 1.9 MPa at 800 °C. When the holding time was extended to 3 h, the corresponding ranges decreased to 1.2–2.0, 0.9–1.5, and 0.6–1.1 MPa, respectively. These results demonstrate that both thermal intensity and exposure duration progressively impaired the tensile load-transfer capacity of the mortar.

The adverse effect of holding time became increasingly pronounced at higher temperatures. For PP_70_, extending the holding time from 1 to 3 h reduced the tensile bond strength from 2.4 to 2.0 MPa at 600 °C, from 2.2 to 1.5 MPa at 700 °C, and from 1.9 to 1.1 MPa at 800 °C. Thus, prolonged exposure caused only a moderate additional reduction at 600 °C but substantially accelerated bond deterioration at 700 and 800 °C.

PP replacement had a noticeable influence on bond-strength retention. Among the PP-containing mixtures, PP_70_ generally exhibited the highest residual bond strength, whereas further increases in PP content led to progressively lower values. Under the most severe condition of 800 °C for 3 h, PP_70_ retained approximately 1.1 MPa, while PP_90_ and PP_100_ retained only about 0.7 MPa. Therefore, the favorable performance of PP_70_ cannot be attributed solely to its ambient-temperature strength, but also reflects its greater resistance to thermally induced bond degradation.

The direct tensile strength showed greater thermal sensitivity than the compressive strength. This difference is mechanically reasonable because compressive load can still be transferred through contact and friction between partially intact solid regions, whereas direct tensile resistance is more sensitive to discontinuities such as pores and thermally induced microcracks [31]. For PP_70_ within the high-volume series, the more homogeneous distribution of the solid phases may reduce local stiffness discontinuities and suppress stress concentration within the bonded region. In contrast, increasing PP replacement to 80–100% produces a more powder-dominated structure with fewer rigid aggregate constraints. This increases the likelihood of crack localization and facilitates the formation of a continuous fracture path under tensile loading. Consequently, PP_70_ achieved the most favorable bond-strength retention, although severe degradation remained unavoidable after prolonged exposure at 800 °C.

### 3.6. Correlation and Temperature Sensitivity

**Figure 8:**
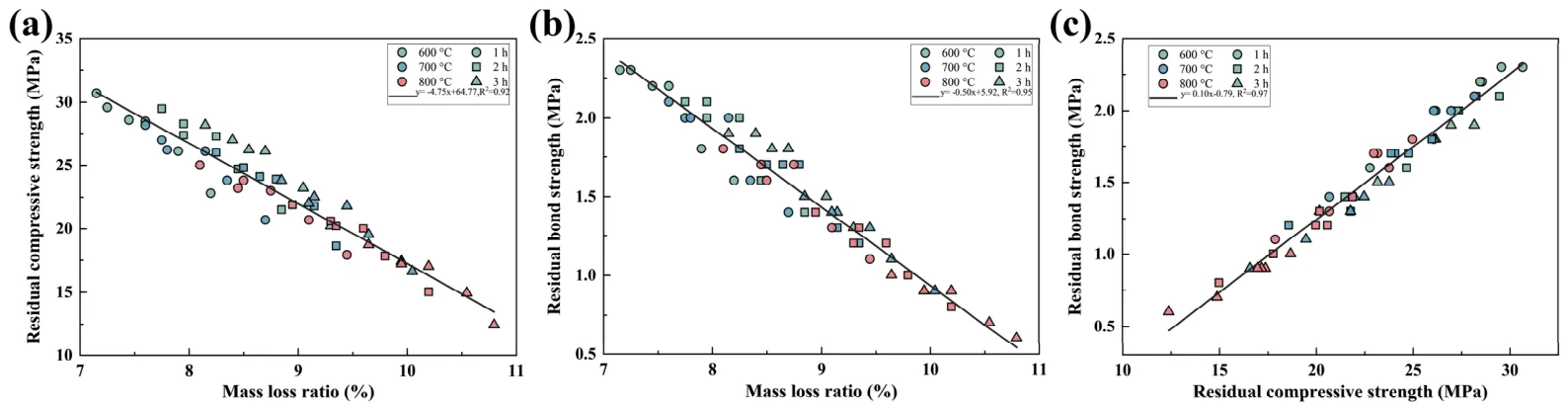
Correlations among the thermal-mechanical properties of PP-GRM: (**a**) mass loss ratio versus residual compressive strength; (**b**) mass loss ratio versus residual bond strength; and (**c**) residual compressive strength versus residual bond strength.

As shown in Figure 8, the residual mechanical properties were strongly correlated with the mass loss ratio. The residual compressive strength and residual bond strength decreased linearly with increasing mass loss ratio, with *R^2^* values of 0.92 and 0.95, respectively. Within the investigated range, each percentage-point increase in mass loss ratio corresponded to reductions of approximately 4.75 MPa in compressive strength and 0.50 MPa in bond strength. A stronger positive correlation was obtained between the two residual strengths (*R^2^* = 0.97), indicating that deterioration of the bulk load-bearing capacity was generally accompanied by degradation of the bonding performance. Although the pooled regressions show co-variation among the measured properties, exposure temperature is a common driver of mass loss and residual strength. Therefore, the relatively high *R*^2^ values may partly arise from their shared temperature dependence, together with the effects of holding time and PP replacement level. These pooled fits are used only as descriptive summaries and should not be interpreted as independent or causal relationships.

**Figure 9:**
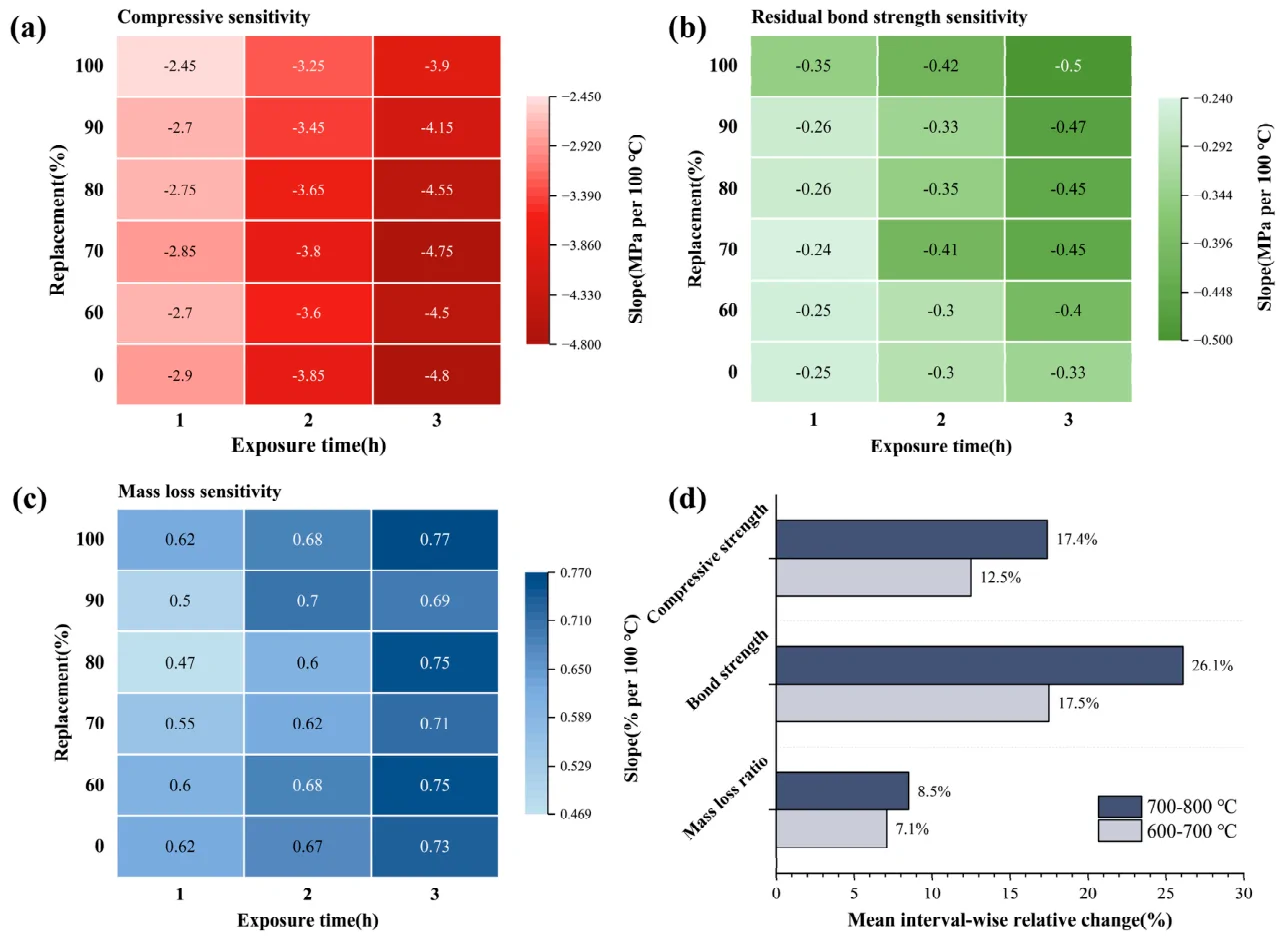
Temperature-sensitivity analyses of PP-GRM: (**a**) residual compressive strength; (**b**) residual bond strength; (**c**) mass loss ratio; and (**d**) mean interval-wise relative changes over 600–700 and 700–800 °C.

The sensitivity maps in Figure 9 demonstrate that exposure duration was a dominant factor governing the temperature response. The absolute compressive-strength sensitivity increased consistently from 1 to 3 h for all mixtures. For PP_0_, the compressive strength sensitivity changed from −2.90 to −4.80 MPa per 100 °C as the holding time increased from 1 to 3 h, whereas PP_100_ changed from −2.45 to −3.90 MPa per 100 °C. A different tendency was observed for bond strength. After prolonged exposure, the PP-containing mixtures generally exhibited larger residual bond strength sensitivities than the control, with the 100% replacement mixture reaching −0.50 MPa per 100 °C after 3 h. This divergence indicates that improved stability of the bulk compressive response did not necessarily ensure preservation of the bond performance under severe thermal exposure. The mass loss ratio sensitivity also generally increased with exposure duration, but its variation with PP replacement was non-monotonic. Replacement levels of 70–90% produced relatively low sensitivities under several exposure conditions, whereas the 100% PP mixture reached the highest value of 0.77% per 100 °C after 3 h. Thus, the thermal benefit of PP depended on both the evaluated property and exposure duration, rather than increasing continuously with replacement level.

As shown in Figure 9d, the mean relative changes during 700–800 °C were consistently greater than those during 600–700 °C. The changes increased from 12.5% to 17.4% for compressive strength, from 17.5% to 26.1% for tensile bond strength, and from 7.1% to 8.5% for mass loss ratio. Bond strength exhibited the largest interval-wise change, identifying the tensile bond strength response as the most temperature-sensitive property. Overall, the results indicate an accelerated deterioration tendency above 700 °C and reveal a trade-off between compressive-strength retention and tensile bond strength stability at high PP replacement levels.

### 3.7. Microstructural Evolution

**Figure 10:**
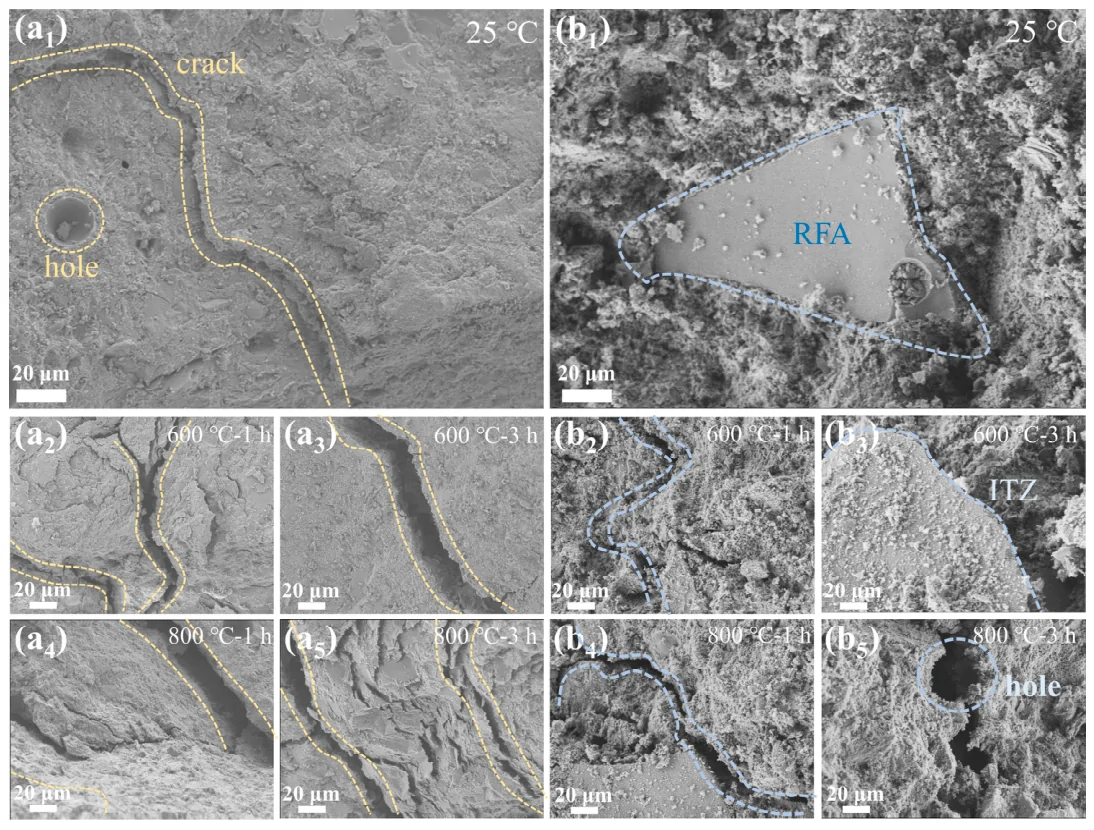
Secondary-electron (SE) SEM micrographs of (**a**) PP_0_ and (**b**) PP_70_ under different thermal exposure conditions: (1) 25 °C; (2) 600 °C for 1 h; (3) 600 °C for 3 h; (4) 800 °C for 1 h; (5) 800 °C for 3 h.

To provide mechanistic insight into the macroscopic results presented in Section 3.1, Section 3.2, Section 3.3, Section 3.4, Section 3.5 and Section 3.6, PP_0_ and PP_70_ were selected to compare the unmodified reference with the PP-containing mixture that exhibited the most favorable overall residual mechanical performance. SEM analyses were therefore conducted on these two mixtures to clarify how PP incorporation influenced the thermal damage process at the microstructural levels. Figure 10 compares the microstructural evolution of PP_0_ and PP_70_ before and after thermal exposure. At 25 °C, the PP_0_ matrix exhibited a relatively continuous morphology, although an isolated hole and a pre-existing microcrack were visible (Figure 10a_1_). In PP_70_, RFA was closely surrounded by the geopolymer matrix, and no continuous interfacial gap was observed (Figure 10b_1_). This morphology indicates that PP_70_ maintained effective contact between the RFA and the surrounding reaction products.

After 1 h at 600 °C, the matrix continuity was largely retained, but the cracks became more open (Figure 10a_2_), consistent with contraction of the geopolymer matrix during the removal of bound water [13]. Prolonging the exposure to 3 h further widened the cracks and caused local fragmentation of the surrounding matrix (Figure 10a_3_). These observations indicate that the damage at 600 °C developed progressively with exposure duration rather than through an abrupt loss of matrix integrity. In comparison, the RFA-matrix contact in PP_70_ was still largely maintained after 1 h at 600 °C (Figure 10b_2_). However, after 3 h, a more distinguishable and porous ITZ developed around the RFA (Figure 10b_3_), suggesting that thermal damage was increasingly concentrated at the interface.

At 800 °C, the two mixtures exhibited different dominant damage patterns. PP_0_ showed wide fissures and a flaky morphology after 1 h (Figure 10a_4_), followed by more extensive cracking and local fragmentation after 3 h (Figure 10a_5_). These features indicate gel-network collapse and loss of matrix continuity. By contrast, PP70 displayed pronounced local separation around the RFA after 1 h at 800 °C (Figure 10b_4_). After 3 h, cavity and enlarged voids were observed (Figure 10b_5_), demonstrating deterioration of the RFA-matrix ITZ. The progressive interfacial opening is consistent with the cumulative effects of dehydration and differential thermal deformation [14].

PP incorporation altered the thermal-damage mode. The micrographs suggest that damage in PP_70_ became increasingly concentrated near the RFA–matrix ITZ. The comparatively less continuous cracking of the PP_70_ matrix helps explain its relatively improved compressive strength stability under some exposure conditions. However, the pronounced degradation of the ITZ at prolonged high temperatures shows that preservation of the bulk matrix does not necessarily ensure interfacial stability. This observation supports the divergence identified in Section 3.6 between compressive-strength sensitivity and bond-strength sensitivity.

### 3.8. Phase Assemblage and Thermal Evolution

**Figure 11:**
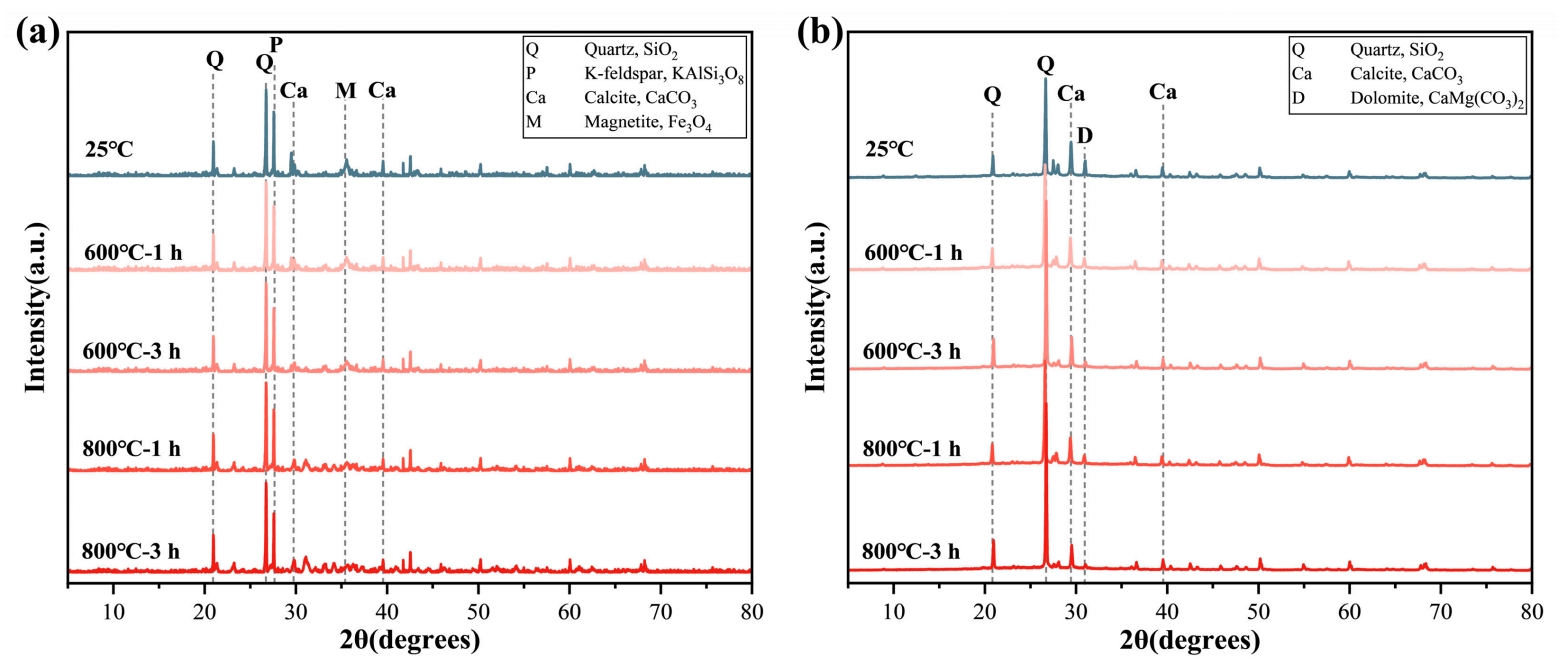
XRD patterns of (**a**) PP_0_ and (**b**) PP_70_ under different thermal exposure conditions.

The XRD patterns of PP_0_ and PP_70_ were examined to determine whether the thermal damage observed at the macro- and microscales was accompanied by changes in phase assemblage (Figure 11). At 25 °C, PP_0_ was characterized by quartz, K-feldspar, calcite, and magnetite, whereas quartz, calcite, and dolomite were identified in PP_70_. These sharp reflections were mainly associated with the crystalline constituents inherited from the raw materials and recycled aggregate.

After exposure to 600 °C, the positions and overall distribution of the principal diffraction peaks remained essentially unchanged in both mixtures, irrespective of exposure duration. The loss of physically bound and structural water from the aluminosilicate gel, followed by further condensation of the silanol and aluminol groups, induced shrinkage of the binding network. The reversible α- to β-quartz transformation occurring during heating may have additionally intensified the thermal incompatibility between the crystalline particles and the surrounding matrix [26]. This interpretation is consistent with the progressive crack widening observed in Figure 10a_2_,a_3_ and the moderate reduction in residual mechanical properties at 600 °C. More discernible changes occurred after exposure to 800 °C. Calcite- and dolomite-related reflections remained detectable, although their relative intensities varied with thermal exposure. Partial decarbonation may have occurred at elevated temperature, particularly during prolonged exposure at 800 °C. In PP_0_, several weak reflections became increasingly resolved, particularly within the *2θ* range of approximately 30–40°, and this tendency was more evident after 3 h. These features indicate an increase in the structural ordering of the dehydrated aluminosilicate matrix and the onset of crystallization.

Compared with PP_0_, the phase assemblage of PP_70_ varied less noticeably with increasing temperature and exposure duration. No clearly identifiable new crystalline phase was observed within the detection limit of the XRD measurements, suggesting that extensive crystallization was unlikely under the investigated conditions. However, the content of the amorphous or glassy aluminosilicate fraction cannot be quantified from the present qualitative XRD patterns. When considered together with the SEM and residual-strength results, the comparatively limited variation in the PP_70_ diffraction profiles indicates a relatively stable detectable phase assemblage.

The XRD and SEM results reveal a two-stage thermal evolution mechanism. At 600 °C, degradation was dominated by gel dehydration and thermally induced incompatibility without substantial alteration of the crystalline phase assemblage. At 800 °C, further aluminosilicate-network rearrangement occurred concurrently with matrix cracking and interfacial separation. PP incorporation therefore improved the stability of the bulk matrix to some extent but did not eliminate interface-controlled damage under prolonged high-temperature exposure.

### 3.9. Embodied Carbon Assessment

A simplified cradle-to-gate embodied-carbon assessment was conducted using 1 m^3^ of PP-GRM as the functional unit. Because the present analysis focused on the comparative effect of PP replacement, only emissions associated with raw-material production were considered, whereas transportation, mixing, curing, and elevated-temperature testing were excluded [32]. The carbon emission factors adopted in the calculation are summarized in Table 4. The adopted emission factors are literature-based production-stage values rather than supplier-specific environmental product declarations. Consequently, the calculated embodied-carbon values should be interpreted as comparative estimates among the investigated mixtures rather than as product-specific life-cycle inventories. The embodied carbon of each mixture was calculated as:

(7) $$C_{j}=\sum_{i=1}^{n}m_{i,j}EF_{i}$$

where *C_j_* is the embodied carbon of mixture *j* (kg CO_2_-eq/m^3^), *m_i, j_* is the dosage of material *i* (kg/m^3^), and *EF_i_* is its corresponding emission factor (kg CO_2_-eq/kg).

To account for differences in mechanical performance, the strength-normalized carbon intensity was calculated as:

(8) $$CI_{T,j}=\frac{C_{j}}{f_{c,T,j}}$$

where *CI_T,j_* is the strength-normalized carbon intensity of mixture *j* under exposure condition *T* (kg CO^2^-eq/(m^3^⋅MPa)); *C_j_* is the embodied carbon of mixture *j* (kg CO_2_-eq/m^3^); *f_c,T,j_* is the compressive strength at ambient temperature or the residual compressive strength after exposure to 800 °C for 3 h (MPa).

As shown in Table 5, the embodied carbon increased substantially with increasing PP replacement [38]. The embodied carbon increased from 347.4 kg CO_2_-eq/m^3^ for PP_0_ to 792.9 kg CO_2_-eq/m^3^ for PP_100_, corresponding to an increase of 128.3%. PP_70_ exhibited an embodied carbon of 659.3 kg CO_2_-eq/m^3^, which was 89.8% higher than that of PP_0_ [39]. This pronounced increase resulted from the replacement of low-emission RFA with a substantially larger mass of commercially processed PP when the substitution was performed on an equal absolute-volume basis [40].

After exposure to 800 °C for 3 h, PP_70_ exhibited the lowest calculated carbon intensity among the PP-modified mixtures, with a *CI*_800-3 h_ value of 35.07 kg CO_2_-eq/(m^3^·MPa), compared with 35.74 kg CO_2_-eq/(m^3^·MPa) for PP_60_. This difference is only approximately 1.4%. Given the experimental variability of the residual strength and the uncertainty associated with literature-based emission factors, such a small difference should not be interpreted as a robust environmental advantage of PP_70_ over PP_60_. PP_0_ remained the most carbon-efficient mixture under both ambient and elevated-temperature conditions. Accordingly, PP_70_ should be regarded only as the lowest calculated carbon-intensity option among the PP-modified mixtures under the specific condition of 800 °C for 3 h.

## 4. Conclusions

This study investigated the effects of volumetrically replacing RFA with PP on the ambient-temperature properties, high-temperature resistance, thermal-damage mechanisms, and embodied carbon of GRM. The main conclusions are as follows.

(1) The effect of PP was governed by a balance between matrix densification and the reduction in the aggregate skeleton. Moderate replacement (60–70%) improved particle packing and enhanced the accommodation of vapor transport and thermal deformation. Among the investigated PP-containing mixtures, PP_70_ showed a comparatively favorable overall mechanical response. It exhibited an ambient compressive strength of 31.7 MPa. After exposure to 800 °C for 3 h, PP_70_ retained approximately 18.8 MPa, corresponding to 59.3% of its ambient compressive strength.
(2) Thermal deterioration was governed primarily by exposure temperature and was intensified by prolonged holding. Deterioration accelerated between 700 and 800 °C, and tensile bond strength showed a greater temperature sensitivity than compressive strength. The pooled correlations among mass loss and residual strengths describe their overall co-variation, although their high R^2^ values may partly reflect their common dependence on exposure temperature.
(3) The thermal response followed a two-stage damage mechanism. At 600 °C, degradation was dominated by dehydration, gel contraction, and thermal incompatibility, with no substantial change in the principal crystalline phases. At 800 °C, further aluminosilicate-network rearrangement occurred together with crack propagation and interfacial separation. Pumice powder reduced continuous matrix cracking to some extent, but prolonged high-temperature exposure shifted the dominant damage toward deterioration of the ITZ.
(4) The mechanical benefits of pumice powder were accompanied by an embodied-carbon penalty. The embodied carbon increased from 347.4 kg CO_2_-eq/m^3^ for PP_0_ to 792.9 kg CO_2_-eq/m^3^ for PP_100_ because low-emission RFA was replaced by commercially processed pumice powder. PP_70_ exhibited the lowest strength-normalized carbon intensity among the PP-containing mixtures after exposure to 800 °C for 3 h. Therefore, within the investigated high-volume replacement range of 60–100%, PP_70_ represents a comparatively favorable compromise when high-temperature mechanical stability is prioritized.

## Figures and Tables

**Table 1:** Chemical compositions of MK and PP (wt.%).

Name	SiO_2_	Al_2_O_3_	Fe_2_O_3_	MgO	K_2_O	CaO	LOI
MK	59.50	32.83	5.43	0.32	0.76	0.22	0.94
PP	73.80	16.52	1.90	0.10	4.10	0.35	3.23

**Table 2:** Physical properties of MK and PP.

	Specific Surface Area (m^2^/g)	Apparent Density (kg/m^3^)	Thermal Conductivity W/(m·K)
MK	15.133	2420	0.28
PP	1.915	2490	0.12

**Table 3:** Mix proportions of PP-GRM (kg/m^3^).

Sample	Replacement Rate	PP	MK	RFA	Pre-Wetting Water	Alkali-Activator		
						NaOH	Na_2_SiO_3_	Distilled Water
PP_0_	0%	-	520.0	1237.5	116.3	40.0	245.0	75.0
PP_60_	60%	813.4	520.0	495.4	46.6	40.0	245.0	75.0
PP_70_	70%	948.9	520.0	371.3	34.9	40.0	245.0	75.0
PP_80_	80%	1084.5	520.0	247.5	23.3	40.0	245.0	75.0
PP_90_	90%	1220.1	520.0	123.8	11.6	40.0	245.0	75.0
PP_100_	100%	1355.6	520.0	-	-	40.0	245.0	75.0

**Table 4:** Carbon emission factors adopted for the raw materials used in PP-GRM.

Material	Abbreviation	Carbon Emission Factor (kg CO_2_-eq/kg)
Metakaolin	MK	0.330 [33]
Recycled fine aggregate	RFA	0.00584 [34]
Pumice powder	PP	0.334 [35]
Sodium hydroxide	NaOH	1.580 [36]
Sodium silicate solution	SS	0.430 [37]

**Table 5:** Embodied carbon and strength-normalized carbon intensity of PP-GRM.

Mixture	Embodied Carbon (kg CO_2_-eq/m^3^)	Change Relative to PP_0_ (%)	*CI*_25_	*CI*_800-3 h_
PP0	347.4	0.0	10.79	19.85
PP60	614.7	+77.0	20.42	35.74
PP70	659.3	+89.8	20.80	35.07
PP80	703.8	+102.6	23.23	41.40
PP90	748.4	+115.4	26.08	50.23
PP100	792.9	+128.3	30.62	63.43

## Data Availability

The original contributions presented in this study are included in the article. Further inquiries can be directed to the corresponding authors.

## References

[1] McLellan B.C., Williams R.P., Lay J., van Riessen A., Corder G.D. (2011). Costs and Carbon Emissions for Geopolymer Pastes in Comparison to Ordinary Portland Cement. J. Clean. Prod..

[2] Miao C., Mu S. (2025). Development and Prospect of Concrete Technology in China. Mater. Rep. Solidwaste Ecomater..

[3] Huang J., Xu X., Alam K.R., Wu Y., Qu C., Wang J. (2026). Sustainable Recycling of Phosphogypsum and Recycled Concrete Aggregate into Base Course for Pavement: Mix Design and Durability Evaluation. Mater. Rep. Solidwaste Ecomater..

[4] Snellings R., Suraneni P., Skibsted J. (2023). Future and Emerging Supplementary Cementitious Materials. Cem. Concr. Res..

[5] de Castro Carvalho I., Zhang Z., Kirchheim A.P., Costa H.N., Cabral A.E.B. (2025). Waste Clay Brick Utilization in Alkali-Activated Materials: A Review on Fresh and Hardened State Properties. Mater. Rep. Solidwaste Ecomater..

[6] Yang J., Du P., Cheng X. (2025). Advances and Prospects in Rapid Curing Technology for Geopolymers. Mater. Rep. Solidwaste Ecomater..

[7] Nuaklong P., Sata V., Chindaprasirt P. (2016). Influence of Recycled Aggregate on Fly Ash Geopolymer Concrete Properties. J. Clean. Prod..

[8] Hasnaoui A., Ghorbel E., Wardeh G. (2021). Performance of Metakaolin/Slag-Based Geopolymer Concrete Made with Recycled Fine and Coarse Aggregates. J. Build. Eng..

[9] Şahin F., Uysal M., Canpolat O., Aygörmez Y., Cosgun T., Dehghanpour H. (2021). Effect of Basalt Fiber on Metakaolin-Based Geopolymer Mortars Containing Rilem, Basalt and Recycled Waste Concrete Aggregates. Constr. Build. Mater..

[10] Ma T., Liu H., Shen X., Zhu P., Xing Z., Li Y., Dai X. (2026). A Cross-Scale Investigation into the Thermal Degradation Pathways of Sustainable Geopolymer Recycled Mortar Modified by Carbon-Sequestering Biochar. Constr. Build. Mater..

[11] Manzoor T., Bhat J.A., Shah A.H. (2024). Performance of Geopolymer Concrete at Elevated Temperature—A Critical Review. Constr. Build. Mater..

[12] Zhang W., Zhang Z., Yin Y., Shi C., Zhu Y., Zhu H., Jiang Z. (2025). A Review of Geopolymers for High-Temperature and Fireproof Applications. Mater. Rep. Solidwaste Ecomater..

[13] Bayer Öztürk Z., Atabey İ.İ. (2022). Mechanical and Microstructural Characteristics of Geopolymer Mortars at High Temperatures Produced with Ceramic Sanitaryware Waste. Ceram. Int..

[14] Kai M.-F., Dai J.-G. (2021). Understanding Geopolymer Binder-Aggregate Interfacial Characteristics at Molecular Level. Cem. Concr. Res..

[15] Zhang H.Y., Kodur V., Wu B., Yan J., Yuan Z.S. (2018). Effect of Temperature on Bond Characteristics of Geopolymer Concrete. Constr. Build. Mater..

[16] Ma T., Liu H., Chen C., Yang L., Guo X., Jiang F., Zhu P. (2026). Fly Ash Hollow Microspheres Modulating the Thermal Transport Behavior and Mechanism in Geopolymer Recycled Mortar: Variations in Mechanical Properties, Pore Structure, and Phase Composition. Ceram. Int..

[17] Kabay N., Miyan N., Özkan H. (2021). Utilization of Pumice Powder and Glass Microspheres in Cement Mortar Using Paste Replacement Methodology. Constr. Build. Mater..

[18] Safari Z., Kurda R., Al-Hadad B., Mahmood F., Tapan M. (2020). Mechanical Characteristics of Pumice-Based Geopolymer Paste. Resour. Conserv. Recycl..

[19] Dener M., Karatas M., Mohabbi M. (2021). Sulfate Resistance of Alkali-Activated Slag/Portland Cement Mortar Produced with Lightweight Pumice Aggregate. Constr. Build. Mater..

[20] (2025). Fire-Resistance Tests—Elements of Building Construction—Part 1: General Requirements.

[21] (2009). Standard for Test Method of Performance on Building Mortar.

[22] Sun B., Ye G., de Schutter G. (2022). A Review: Reaction Mechanism and Strength of Slag and Fly Ash-Based Alkali-Activated Materials. Constr. Build. Mater..

[23] Chen S., Ruan S., Zeng Q., Liu Y., Zhang M., Tian Y., Yan D. (2022). Pore Structure of Geopolymer Materials and Its Correlations to Engineering Properties: A Review. Constr. Build. Mater..

[24] Zhao J., Wang S., Wang Z., Wang K., Fu C. (2023). Bond Performance between FRP Bars and Geopolymer Concrete after Elevated Temperature Exposure. Constr. Build. Mater..

[25] Nikolov A., Karamanov A. (2022). Thermal Properties of Geopolymer Based on Fayalite Waste from Copper Production and Metakaolin. Materials.

[26] Babalola O.E., Awoyera P.O., Le D.-H., Bendezú Romero L.M. (2021). A Review of Residual Strength Properties of Normal and High Strength Concrete Exposed to Elevated Temperatures: Impact of Materials Modification on Behaviour of Concrete Composite. Constr. Build. Mater..

[27] Klima K.M., Schollbach K., Brouwers H.J.H., Yu Q. (2022). Thermal and Fire Resistance of Class F Fly Ash Based Geopolymers—A Review. Constr. Build. Mater..

[28] Zulkifly K., Cheng-Yong H., Yun-Ming L., Bayuaji R., Abdullah M.M.A.B., Ahmad S.B., Stachowiak T., Szmidla J., Gondro J., Jeż B. (2021). Elevated-Temperature Performance, Combustibility and Fire Propagation Index of Fly Ash-Metakaolin Blend Geopolymers with Addition of Monoaluminium Phosphate (MAP) and Aluminum Dihydrogen Triphosphate (ATP). Materials.

[29] Xie J., Li J., Zhang B., Chen W., Zhong H., Yang J., Yu T., Feng Y. (2024). Effects of Pretreated Recycled Fine Aggregates on the Mechanical Properties and Microstructure of Alkali-Activated Mortar. Case Stud. Constr. Mater..

[30] Mustapa N.B., Ahmad R., Ibrahim W.M.W., Abdullah M.M.A.B., Wattanasakulpong N., Nemeș O., Sandu A.V., Vizureanu P., Sandu I.G., Kartikowati C.W. (2023). Effect of Sintering Mechanism towards Crystallization of Geopolymer Ceramic—A Review. Materials.

[31] Vijaya Prasad B., Anand N., Kanagaraj B., Kiran T., Lubloy E., Naser M.Z., Arumairaj P.D., Andrushia D. (2023). Investigation on Residual Bond Strength and Microstructure Characteristics of Fiber-Reinforced Geopolymer Concrete at Elevated Temperature. Case Stud. Constr. Mater..

[32] Valente M., Sambucci M., Chougan M., Ghaffar S.H. (2022). Reducing the Emission of Climate-Altering Substances in Cementitious Materials: A Comparison between Alkali-Activated Materials and Portland Cement-Based Composites Incorporating Recycled Tire Rubber. J. Clean. Prod..

[33] Aldawish A., Kulasegaram S. (2026). Sustainability-Focused Evaluation of Self-Compacting Concrete: Integrating Explainable Machine Learning and Mix Design Optimization. Appl. Sci..

[34] Lei B., Yu L., Chen Z., Yang W., Deng C., Tang Z. (2022). Carbon Emission Evaluation of Recycled Fine Aggregate Concrete Based on Life Cycle Assessment. Sustainability.

[35] Zada N.S., Shafiq N., Baarimah A.O., Alawag A.M. (2026). Enhancing Sustainability and Mechanical Performance of Lime Based Concrete Using Volcanic Pumice Powder Ash. Discov. Sustain..

[36] Hong J., Chen W., Wang Y., Xu C., Xu X. (2014). Life Cycle Assessment of Caustic Soda Production: A Case Study in China. J. Clean. Prod..

[37] Fawer M., Concannon M., Rieber W. (1999). Life Cycle Inventories for the Production of Sodium Silicates. Int. J. Life Cycle Assess..

[38] Munir Q., Abdulkareem M., Horttanainen M., Kärki T. (2023). A Comparative Cradle-to-Gate Life Cycle Assessment of Geopolymer Concrete Produced from Industrial Side Streams in Comparison with Traditional Concrete. Sci. Total Environ..

[39] Abdellatief M., Hamouda H., Palou M.T., Tawfik T.A. (2025). Synergistic Effects of Basalt Fiber and Volcanic Pumice Powder in High-Strength Geopolymer Concrete. Sci. Rep..

[40] Imtiaz L., Kashif-ur-Rehman S., Alaloul W.S., Nazir K., Javed M.F., Aslam F., Musarat M.A. (2021). Life Cycle Impact Assessment of Recycled Aggregate Concrete, Geopolymer Concrete, and Recycled Aggregate-Based Geopolymer Concrete. Sustainability.

